# Drought in the Southeast: Lessons for Water Management

**DOI:** 10.1289/ehp.116-a168

**Published:** 2008-04

**Authors:** John Manuel

Long spared the persistent droughts that have plagued the western United States this century, the Southeast suddenly finds itself the most rain-starved region of the country. In the face of this threat, policy makers and utility companies are struggling to identify sensible, sustainable options for managing the region’s water. Although there currently is no immediate public health threat posed by the Southeastern drought, it does point to a very real situation in regions around the world that struggle to maintain an adequate supply of potable water.

According to the Intergovernmental Panel on Climate Change report *Climate Change 2007: The Physical Science Basis*, as global temperatures increase due to rising atmospheric concentrations of carbon dioxide, so does evaporation. That, combined with cyclical drought, could pose dire threats to water supplies. By one model, published in volume 78, issue 5 (2006) of the *Journal of Hydrometeorology*, if global warming–related precipitation changes continue apace, the percentage of the Earth’s surface in severe drought could rise from the current 3% to 30% by 2100.

The Southeastern drought has already had serious economic consequences, according to the National Drought Mitigation Center at the University of Nebraska, which estimates in its Winter 2008 *DroughtScape* newsletter that 2007 losses to major field crops including corn, wheat, soybeans, cotton, and hay totaled more than $1.3 billion. Cattle farmers, nursery and landscape businesses, and recreation and tourism also have been hard hit. Low lake levels have forced power companies such as the Tennessee Valley Authority (TVA) and Duke Energy in North Carolina to reduce electricity generation from cheap, renewable hydropower and substitute more expensive and polluting fossil fuels. By the same token, if cooling reservoir levels were to fall far enough, it could force the shutdown of nuclear power plants.

The drought is having political consequences as well, pitting downstream and upstream water users against each other. For example, Alabama and Florida successfully sued Georgia over a state plan for withdrawing water from Lake Lanier, the main source of drinking water for the Atlanta metro region. Lake Lanier feeds the Chattahoochee River, which supplies water to towns in Alabama and Florida and whose flow is key to the survival of a host of endangered species such as freshwater mussels and sturgeon. The three states have feuded since 1989 over how to divide the water, but the drought has exacerbated the problem as the various parties fight over a much-reduced volume of water.

## A Dry Southeast

After an extended dry period stretching back to fall 2005, rains in the winter of 2006–2007 offered some respite to the Southeast. But the fall 2007 arrival of La Niña, a condition that recurs every few years and can persist as long as two years, diverted seasonal rains north and west. The hurricanes and tropical storms that had bailed the region out in past dry summers failed to materialize.

As the drought persisted, political leaders urged citizens to limit their water use. “I encourage all Georgians to make their dry lawns and dirty cars a badge of honor,” said Georgia governor Sonny Perdue in a 25 October 2007 press release. Proactive utilities like North Carolina’s Orange Water and Sewer Authority (OWASA), which enacted year-round conservation requirements after an earlier severe drought in 2002, activated additional restrictions as soon as the potential severity of the current drought became apparent. The Birmingham (Alabama) Water Works imposed a surcharge on about 25,000 of its customers for excess water usage in June.

Georgia’s Environmental Protection Division (EPD) declared a level four drought response for all counties in northern Georgia (an area that includes Atlanta), prohibiting most outdoor residential water use. At the end of October, Perdue directed the Georgia EPD to modify surface water and groundwater withdrawal and drinking water permits to achieve a 10% reduction in water withdrawals in the same region. Georgia is unique among Southeastern states in having statewide permitting authority over entities and companies that withdraw more than 100,000 gallons per day or that operate a drinking water system serving 25 or more people. In contrast, other states may require localities to submit water plans and may require permits for withdrawals in designated areas, but do not exert control statewide.

Winter came. Reservoir levels continued to drop. Groundwater and streams failed to recharge. By mid-February 2008, nearly all of the Southeast remained abnormally dry. Hard-hit water systems began rolling out a host of programs and policies to buttress their pleas for conservation.

## Efforts to Conserve

Conservation measures and programs undertaken by municipalities and utilities generally fall into four categories. The first is mandatory restrictions on certain types of water use. Restrictions on irrigation of residential lawns are one of the most visible and effective ways to conserve water. Raleigh, North Carolina, has also banned car washing except at facilities that comply with a conservation certification program, prohibited filling of new swimming pools, required that water leaks be repaired within 24 hours of notification by the city public utility’s director, prohibited serving drinking water in restaurants unless requested, and directed innkeepers to ask guests to reuse their towels and linens between laundering.

A second category of conservation measures involves giveaways or rebates of water-saving devices, including low-flow showerheads and toilets, faucet aerators, and replacement toilet flappers (warped flappers allow water to leak). A 2004 study by the Tampa Water Department and the U.S. Environmental Protection Agency (EPA), *Tampa Water Department Residential Water Conservation Study*, showed that retrofitting homes with such devices lowered daily water usage by an average of 92 gallons (46%) per household. Stemming leakage resulted in substantial savings of 15 gallons per person per day. According to the study, toilet leaks contribute the most to household leakage.

In 1993 the City of Tampa began offering rebates of up to $100 on low-flow toilets. Toilets installed before 1994 typically use 3.5–7.0 gallons per flush. In 1994, the EPA began requiring all toilets sold in the United States to use no more than 1.6 gallons per flush, although many old toilets remain in use. As of the end of 2005, the city had provided $3 million in rebates to replace 33,765 toilets for an annual savings of 434 million gallons. The program will end in April 2008 due to financial constraints.

Educational programs are a third way to promote conservation. Such programs are widespread and extend from the federal to the local level. The EPA’s WaterSense program, for example, offers tips on how consumers can reduce normal usage by 20%. In Georgia, Cobb County’s Give Them An Inch…Grow A Yard program shows residents how to maintain a healthy lawn using just an inch of water per week (at press time, Cobb County residents were still allowed to hand-water established landscaping on a designated schedule).

Such programs often include a certification component. For example, WaterSense certifies manufacturers and products that comply with EPA specifications for water efficiency. And in Cobb County, local lawncare businesses that offer their customers Give Them An Inch educational materials can become program “partners.”

A fourth means of promoting conservation is the adoption of water rates designed to discourage excessive use. According to Jeff Hughes, director of the Environmental Finance Center (EFC) at the University of North Carolina at Chapel Hill, most utilities in the Southeast historically have relied on declining block rates, meaning they charged lower prices for higher water consumption. That is beginning to change. For example, OWASA adopted a seasonal rate in June 2001, charging more for water use in the summer when demand was higher. In October 2007, the utility switched to a tiered block rate for residential customers starting at $1.98 per thousand gallons for the first 2,999 gallons. The rate for the 6,000- to 10,999-gallon bracket is $5.53 per thousand gallons; in contrast, 378 water utilities sampled in the state by the EFC charge a median price of $2.98 per thousand gallons at that consumption level.

“We went from a seasonal to a tiered structure to provide a positive financial incentive to people who use the least amount of water,” says Ed Holland, planning director for OWASA. “Under the seasonal structure, low users were paying the same unit cost as everybody else. With the tiered structure, they’re rewarded for lower use.”

## Results

Efforts to lower water usage in the Southeast have yielded modest results. Cities including Atlanta, Raleigh, and Durham (North Carolina) have reported declines in consumption of 7–11% over the same period last year. The Georgia EPD announced in a 25 January 2008 press release that the 80 water systems in northern Georgia collectively achieved a savings of 13.3% over the previous year. “The December water use figures are a shining example of water conservation at work,” EPD director Carol A. Couch was quoted as saying.

Following this news and reacting to pressure from various industry and business groups, Georgia has relaxed some of its restrictions on water use. The EPD announced that facilities that return approximately 100% of the water to the source (e.g., to the watershed) will no longer be subject to the 10% reduction requirement. Georgia power plants are no longer included in the water use calculations; because plant water use depends on operation of the power grid to meet electrical needs, Georgians are now being “asked to conserve water by also conserving energy,” according to the press release.

What measures have yielded the most results? Mandatory restrictions on outdoor watering are clearly effective in the short term. “We knock off about three million gallons per day by going to one-day-a-week watering,” says Allan Williams, water resources director for the City of Greensboro (North Carolina). “If we ban all outdoor watering, we knock off about eight million gallons per day.”

Surcharges on excessive water use also appear to work. The Birmingham Water Works reported that consumption declined from an average of 114 million gallons per day to about 95 million gallons per day after initiating its surcharge.

But restrictions and surcharges are temporary measures. Experts say that to reduce water consumption over the long term, utilities need to charge more for water, and they need to charge customers a higher rate the more they use. “I think the tiered rate structure has been the most successful tool in terms of managing demand,” says Williams. “We went to this rate structure in 1998 and have seen residential consumption drop fifteen percent over the last ten years.”

As for public education and hardware giveways, Williams doubts these have much effect. “Until you start poking people in the wallet,” he says, “you won’t change behavior on a permanent basis.”

## Modifications Necessary

Policy makers, citizen groups, academic experts, and utility representatives have suggested a variety of modifications to current practices to help people conserve. Holland says one of the first things needed is a better way for consumers to track their water usage. “A lot of water systems only send out bills every two or three months, and it’s often difficult to decipher how many gallons of water are actually being billed,” he says. “People need timely billing in order to track the effectiveness of their practices, and the bills should be educational.”

Most homes and apartments have water meters with which customers could monitor their daily use, but Holland says these meters are often inconveniently located and hard to read: “First you have to find the meter, then you have to open the lid, watch out for spiders, and then figure out if the numbers represent gallons, thousands of gallons, or hundred-cubic-foot units.” It would be much better for consumers to have real-time meters located in the house. Then, says Holland, “we’d start seeing the ‘Prius effect,’” referring to the reported tendency of car owners to drive more conservatively when they have gauges like the Toyota Prius’s that register real-time fuel consumption.

Hughes says utilities need to charge more for water, and they need to set their rates high enough to cover the real cost of providing that water. “Utilities are starting to realize that their rates are not sufficient to cover maintenance, plan for future needs, and build new supply,” he says. “They are starting to realize they need to adjust their rates more often.”

Sydney Miller, water resources program manager for the Triangle J Council of Governments, a planning group in Research Triangle Park, North Carolina, says federal grants historically provided the money to build local water and sewer infrastructure. Utilities only needed to recover their operating costs. Miller says there was also political pressure to keep water costs low, to make water affordable for those least able to pay. “A lot of factors led us to where we are today, and the utilities are trying to catch up,” he says.

Hughes says annual rate setting is becoming more common, explaining, “Some systems were not raising rates but once every fifteen years. We promote smaller, more moderate increases on a regular basis.” At the same time, he says, short-range revenue shortfalls have become a major problem for many communities. There are many fixed costs involved in providing water treatment, he says, and a utility that suddenly sells 25% less water will not see its costs go down 25%. Many may see hardly any decrease in cost, and as a result, they often have to respond to sudden consumption drops by increasing prices just so they can stay revenue-neutral. “The public,” he adds, “often does not understand the finances of water production and responds negatively: ‘Is this what we get for conserving?’”

Hughes supports tiered rate structures to promote conservation in many communities, but says these structures must be customized to local circumstances. “Tiered rate structures are not all the same,” he says. “Some are set so high that they shave off excessive uses for a small part of the customer base, but have little impact on encouraging the average user to use water more prudently.”

Others want to see improvements in the way new homes are built. “We need to make sure that new homes have the most efficient irrigation systems, rainwater catchment devices, and indoor appliances,” says Rob Thompson, public interest advocate for the North Carolina Public Interest Research Group (NCPIRG). “That will require changes to the building codes.” The potential for consequent increased costs could be a concern for homebuilders and realty groups, says Thompson, adding that these groups have opposed such increases in the past.

Thompson also wants to see states do more to rein in agricultural use of water. “In North Carolina, agricultural users are only required to report withdrawals of water in excess of one million gallons per day,” he says; agriculture earns a reporting exemption because food production is a vital activity. “Other [nonagricultural] users must report withdrawals above a hundred thousand gallons per day.”

Burgeoning population growth in the Southeast—a 20% increase between 1999 and 2000 alone, according to an article in the 18 October 2007 issue of *BusinessWeek*—has put a strain on local water supplies during dry periods. Some policy makers and analysts are beginning to call for constraints on that growth. “Most of the blame [for water shortages] at the moment is falling squarely on historically low rainfall,” states the *BusinessWeek* article. “But an equally important culprit has been the unbridled growth in the Southeast in the past 50 years [where the] abundance of cheap water has long fueled development.”

*The Christian Science Monitor* reported on 4 February 2008 that Paulding County, Georgia, whose population swelled by 49% from 2000 to 2006, froze rezoning applications in October 2007, fearing that new construction would further strain dwindling water supplies. In Raleigh, the debate over growth is fierce. City council member Thomas Crowder called on the city to temporarily raise the fees it charges for water connections and to consider adopting “water capacity impact fees” to offset future utility costs. That spawned a protest from fellow councilman Philip Isley. “This is a *de facto* growth moratorium,” Isley was quoted as saying in a 1 February 2008 story in the Raleigh *News & Observer*. Within weeks, however, another city council member, Rodger Koopman, proposed a *de jure* moratorium, which city mayor Charles Meeker has said he opposes on the grounds it could put the city’s economy into recession.

Meanwhile, North Carolina’s environmental leaders have started a year-long study of the state’s water supplies and policies to determine if state regulators should play a larger role in decisions about allocating water among local communities. “The population growth alone is going to make it impossible for us to assume we can always count on water for all purposes for all times,” Senator Daniel Clodfelter, cochairman of North Carolina’s Environmental Review Commission, told the *News & Observer*. “There is going to be competition for water in the future. We want to take a look over the horizon and see what kind of procedures we need to make sure we don’t end up in water wars.”

## Figures and Tables

**Figure:**
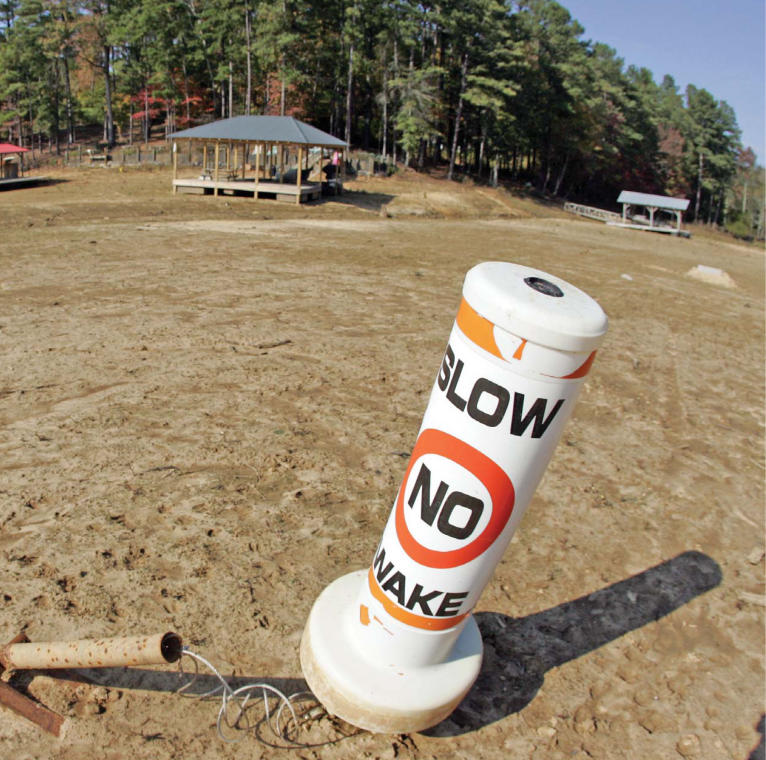
Prolonged drought has shut down recreational activity at Lake Allatoona in Acworth, Georgia, as shown in this 2 November 2007 photo.

